# Uniportal endoscopic decompression without fusion for grade II–III degenerative spondylolisthesis in the elderly: a case series

**DOI:** 10.1093/jscr/rjaf642

**Published:** 2025-08-27

**Authors:** Edwin H Y Lui, Ralph J Mobbs

**Affiliations:** Faculty of Medicine and Health, University of New South Wales, Barker St, Kensington, 2052, NSW, Australia; NeuroSpine Surgery Research Group, Barker St, Randwick, 2031, NSW, Australia; Faculty of Medicine and Health, University of New South Wales, Barker St, Kensington, 2052, NSW, Australia; NeuroSpine Surgery Research Group, Barker St, Randwick, 2031, NSW, Australia; Department of Neurosurgery, Prince of Wales Hospital, Barker St, Randwick, 2031, NSW, Australia; NeuroSpine Clinic, Prince of Wales Private Hospital, Barker St, Randwick, 2031, NSW, Australia

**Keywords:** degenerative spondylolisthesis, uniportal endoscopic decompression, minimally invasive spine surgery, neurogenic claudication, elderly, fusion alternatives

## Abstract

Higher-grade degenerative lumbar spondylolisthesis in the elderly presents a significant management challenge. Standard treatment often involves surgical fusion, which provides stability but carries substantial morbidity in this vulnerable population. Decompression alone is less invasive but lacks strong evidence for these specific grades. This case series reports on facet-sparing uniportal endoscopic decompression as a less invasive alternative in three carefully selected elderly patients (>75 years old) with stable Grade II/III degenerative slips and neurogenic symptoms. All patients experienced rapid postoperative recovery, were discharged the same day and had minimal complications. At 6- to 18-months follow-up, they demonstrated significant symptom relief and functional gains while maintaining radiographic stability without slip progression. These preliminary results suggest uniportal endoscopic decompression alone may be a viable option for selected elderly patients with stable Grade II-III spondylolisthesis and warrants further investigation.

## Introduction

Degenerative spondylolisthesis, characterized by anterior translation of a vertebral body, is a common spinal disorder affecting a significant proportion of the elderly population [1]. Prevalence is estimated at 17% in community-dwelling adults and rises to 25% in women aged ≥70 years [1]. Clinically significant slips are graded using the Meyerding classification, where Grade II corresponds to 25%–50% anterior translation typically resulting from facet and disc degeneration [2]. While instrumented fusion has historically been considered the standard surgical option for symptomatic degenerative spondylolisthesis [3], studies have failed to demonstrate its consistent superiority over decompression alone for disability relief [4]. As fusion is linked to significantly increased peri-operative morbidity, frail elderly patients become poor candidates for such invasive procedures [5].

Endoscopic spine surgery, including the uniportal technique, has gained traction as a minimally invasive alternative offering potential benefits [6]. While endoscopic decompression alone is increasingly supported for stable Grade I degenerative spondylolisthesis [7, 8], evidence remains limited for its use in higher-grade slips. This is particularly relevant in the elderly population, who are traditionally treated with fusion, yet represent a group where less invasive approaches may be especially beneficial [9, 10]. Despite its promise, broader adoption of endoscopic spine surgery is hindered by barriers such as limited training opportunities and insufficient evidence for specific indications like Grade II-III spondylolisthesis [6].

We present three elderly patients over 75 years old with radiographically stable Grade II-III lumbar degenerative spondylolisthesis who underwent facet-sparing uniportal endoscopic decompression without fusion. Notably, all patients achieved rapid recovery, mobilised within 2–3 hours with significantly reduced symptoms without new instability at final follow-up. This case series provides preliminary evidence on the feasibility and early durability of this uniportal endoscopic decompression-only approach in an aging cohort with higher-grade degenerative spondylolisthesis.

## Case series

### Case 1

An 84-year-old woman with comorbidities presented after 6 months of escalating low-back pain, unilateral L5-predominant radiculopathy (visual analogue scale (VAS) 7/10), subjective and objective quality of life (SOQOL) [11] score of 28 (moderate–severe disability), and neurogenic claudication limiting walking to less than 50 m. Previous non-operative therapy including supervised physiotherapy, gabapentin, paracetamol, two fluoroscopic L5/S1 epidural steroid injections were ineffective.

**Figure 1:**
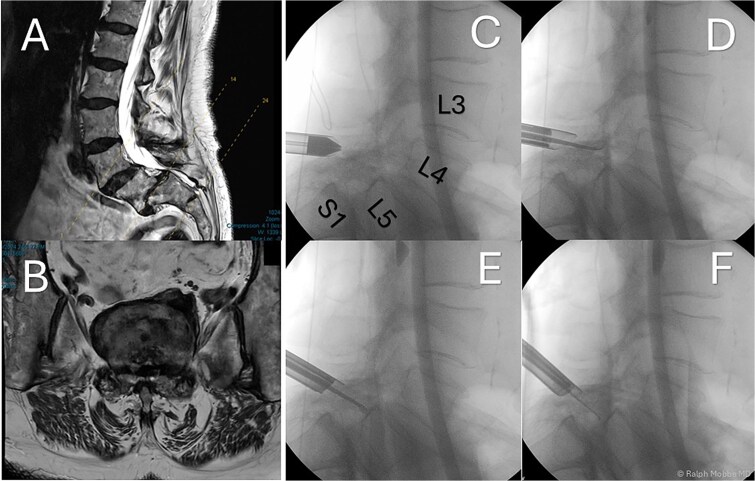
Preoperative and intraoperative imaging of case 1. (A) Sagittal T2-weighted MRI demonstrating grade II degenerative spondylolisthesis at the L5/S1 level. (B) Axial T2-weighted MRI. (C–F) intraoperative fluoroscopic views illustrating the placement of the working channel and guidewire.

Standing radiographs and magnetic resonance imaging (MRI) demonstrated stable Grade-II L5/S1 degenerative spondylolisthesis with severe bilateral foraminal and lateral-recess stenosis compressing the exiting L5 and traversing S1 roots (Fig. 1). Computed tomography (CT) confirmed intact pars and severe facet arthropathy. Because open decompression/fusion was judged high risk due to significant comorbidities, uniportal full-endoscopic decompression alone under conscious sedation was offered.

The operation was performed with patient in the prone position with free abdominal pressure. A skin incision was made positioned just lateral to the midline spinous process at the targeted lumbar interspace. Under fluoroscopic visualization, a series of dilators were advanced through the paraspinal muscles to the lamina, establishing a uniportal working channel (Elliquence, USA) for the endoscopic system with constant irrigation (Fig. 1). Hypertrophied ipsilateral ligamentum flavum and a portion of the ipsilateral facet joint were removed with radiofrequency probe, Kerrison rongeur and grasping forceps, preserving the majority of the facet joint on the approach side.

Free pulsation of the L5 and S1 roots on the symptomatic side was verified endoscopically; no instrumentation was inserted. Operative time was 68 minutes, blood loss <1 cc and no dural tear occurred. She mobilized 3 hours post-op and she was discharged on the same day without analgesics. At 3-month follow-up, she reported VAS 1–2/10, SOQOL score [11] improvement from 28 to 54, and walked unassisted for >1 km. Imaging confirmed maintained alignment without new translation. She remains with symptom reduction and requires no further treatment at final follow up.

### Case 2

A 76-year-old woman presented with 18 months of progressive bilateral L4/5-predominant leg pain (VAS 7–8/10), paraesthesia and intermittent claudication at 40–75 m despite non-steroidal anti-inflammatory drugs (NSAIDs), physiotherapy and a transforaminal injection.

**Figure 2:**
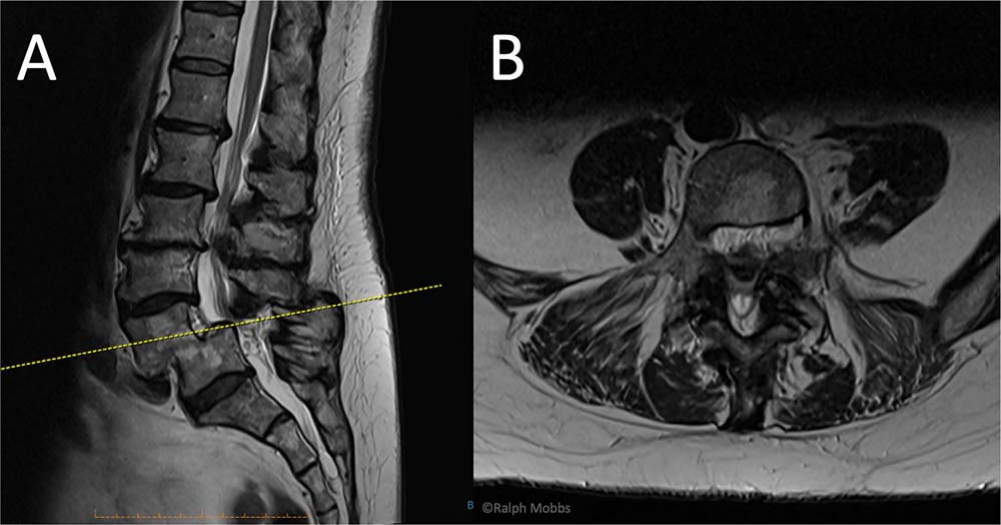
Preoperative MRI of case 2. (A) Sagittal T2-weighted showing L4/L5 spondylolisthesis. (B) Axial T2-weighted.

MRI revealed grade-II L4/5 spondylolisthesis with severe bilateral foraminal and lateral-recess stenosis, a lesser, left-sided L3/4 recess stenosis co-existed (Fig. 2). After shared decision-making, left-sided uniportal endoscopic decompression was performed under general anaesthesia, addressing L4/5. Critical stabilizers, the pars, inter-spinous ligament and majority of facet joints were preserved. No intra-operative complications, nor was instrumentation required.

She ambulated within 4 hours and discharged the evening of surgery. At 8-weeks post intervention, radicular pain had reduced to VAS 3/10 and the SOQOL score [11] improved from 32 to 54. By 3 months, pain was minimal, and she returned to full activities, including 2-3 km daily walks. At 1-year review she remains well with minimal symptoms. No reintervention is planned.

### Case 3

A 78-year-old male with nine months of right L5 radiculopathy (VAS 8/10) and severe neurogenic claudication (<25 m), with failure of conservative measures.

**Figure 3:**
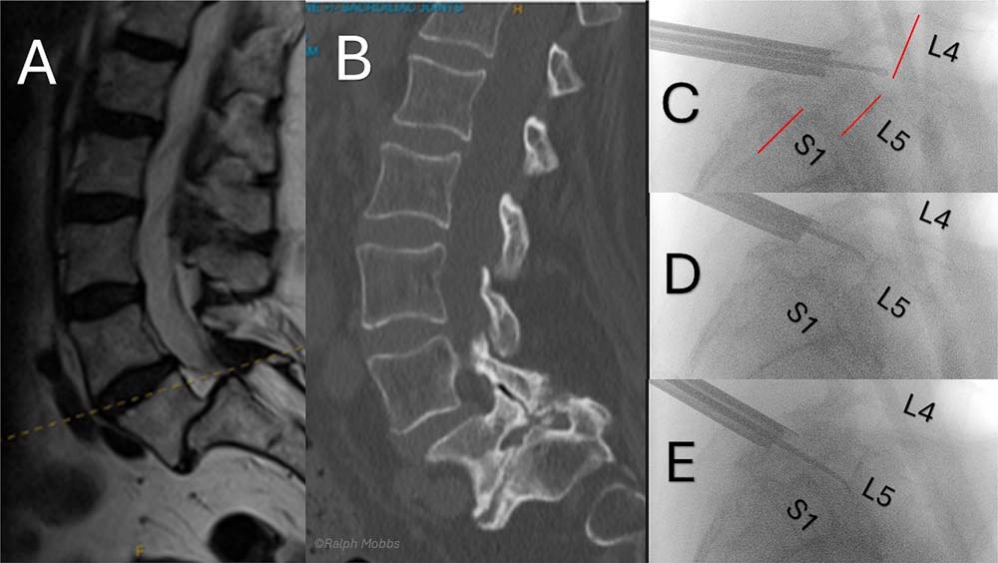
Preoperative and intraoperative imaging of case 3. (A) Sagittal T2-weigthed MRI demonstrating grade II+ degenerative spondylolisthesis at the L5/S1 level. (B) Sagittal CT demonstrating severe foraminal stenosis at L5/S1. (C–E) Intraoperative fluoroscopic images showing placement of the working channel and guidewire using forceps for targeted decompression.

Imaging demonstrated stable borderline Grade III L5/S1 degenerative spondylolisthesis with facet hypertrophy, severe foraminal stenosis and lateral recess narrowing (Fig. 3). Following multidisciplinary review, right interlaminar uniportal endoscopic decompression with conscious sedation was undertaken. The hypertrophic superior and inferior articular process at L5/S1 and flavum were excised with unilateral endoscopic decompression on the symptomatic side, without violating the contralateral facet.

He walked 3 hours post-op and was discharged on the same day. At 6-week review VAS was 2–3/10, with return to work within 2 weeks. At 3 months VAS 1–2/10 and repeat standing radiographs confirmed unchanged slip, with no instability. At the time of this report he remains with significant symptom reduction, uses minimal analgesics and expresses no desire for fusion. Annual surveillance imaging is planned.

## Discussion

This case series suggests that uniportal endoscopic decompression alone may offer a viable alternative to fusion for select elderly patients with Grade II-III degenerative lumbar spondylolisthesis. This is particularly significant, as this population is traditionally considered for higher-morbidity-associated fusion or deemed inoperable. We observed favourable short-term outcomes, including prompt symptom relief and maintained radiographic and clinical stability in all three cases. Based on our experience, an optimal cohort for this approach includes elderly individuals (age > 75 years) with stable listhesis, radiographic intrinsic rigidity and predominant radicular/neurogenic symptoms (VAS ≥ 5/10) without overt instability signs on preoperative and intra-operative assessments. These findings contribute to the ongoing challenge regarding the optimal management of higher-grade slips in vulnerable cohorts [12].

The decision to prioritize direct neural decompression for symptom relief over the assumed biomechanical benefits of fusion in these patients was guided by careful patient selection and the advancements in endoscopic techniques. A core principle underpinning our technique’s ability to maintain stability is the maximal preservation of posterior osseoligamentous structures, specifically aiming for ≥70% of the facet joint surface and pars interarticularis, maintaining the posterior tension band. This meticulous preservation of bone and ligaments is facilitated by the uniportal corridor and angled visualization, enabling targeted bilateral recess decompression with minimal medial fasciectomy. This bone-sparing approach is crucial, as biomechanical studies highlight significantly increased segmental motion and instability risk with medial facet resection exceeding 45%–50% or pars resection over 25% [13]. Unlike more invasive methods risking iatrogenic instability through greater facet sacrifice, our adherence to these preservation principles aims to prevent anterolisthesis progression or new angulation [14].

In contrast to the known peri-operative burdens of instrumented fusion, including substantial operative risks, longer hospital stays, operative duration, greater blood loss, and higher costs [15], our case series highlights the potential for significantly reduced burden with uniportal endoscopic decompression alone. We observed highly favourable peri-operative outcomes, characterized by a mean operative time of 78 minutes, minimal estimated blood loss <1 cc, and same-day discharge in all three cases. These benefits are particularly relevant given the age-dependent increase of major complications following lumbar fusion in the elderly [5].

Importantly, these favourable outcomes were achieved while successfully addressing the primary objective of decompression and maintaining spinal alignment, a goal traditionally sought with instrumented fusion due to concerns about iatrogenic instability from open techniques [16, 17]. Building upon emerging evidence that supports decompression alone as non-inferior to fusion for Grade I spondylolisthesis, particularly in elderly patients who may not derive significant added benefit from fusion [7, 8, 16, 18], our case series extends this concept to Grade II–III stable spondylolisthesis. Our patients experienced notable symptom relief, demonstrated by a significant reduction in VAS scores and substantial functional improvement in SOQOL [11]. Critically, and aligning with a key goal of fusion, we observed no postoperative radiographic or clinical instability, including absence of anterolisthesis progression or new angulation, in all three cases over the 6–18 months of follow-up [14]. This demonstrates that maintaining stability without resorting to fusion may be achievable in carefully selected patients with this technique. This divergence from conventional fusion recommendations for higher grades likely stems from a combination of careful patient selection, comprehensive advanced imaging assessment and the specific bone-sparing capabilities offered by the refined uniportal endoscopic technique [12].

Adding to the factors of careful patient selection and advanced imaging assessment, we note that in this elderly population, advanced degenerative changes are commonly seen with severe facet arthropathy, ligamentous calcification and disc desiccation, often confer significant intrinsic rigidity visible on preoperative imaging. This inherent, age-related stability likely provides sufficient biomechanical support to prevent progressive instability following decompression, even in the presence of higher-grade slips. Accordingly, these characteristics may offer further rationale for considering a non-fusion approach in this specific demographic.

The use of conscious sedation instead of general anaesthesia was a deliberate choice tailored to the elderly, comorbid patient population in this series. This approach aimed to mitigate the elevated risks associated with systemic or spinal anaesthesia in this demographic [19]. Evidence from lumbar decompression literature supports the safety benefits, showing a lower rate of postoperative complications with sedation compared to general anaesthesia [20]. Two patients in our series tolerated conscious sedation well without anaesthesia-related complications. A distinct advantage was the provision of real-time neurological feedback during endoscopic manipulation, which we believe enhanced nerve root safety. This, combined with the procedure’s minimal invasiveness, contributed to rapid recovery and enabled ambulation within hours.

Based on our findings, a pragmatic decision framework for selecting candidates for decompression alone in higher-grade degenerative spondylolisthesis emerges. Key steps include: (i) confirming that predominant neurogenic claudication is the primary source of disability; (ii) assessing for objective clinical and radiographic stability and sufficient intrinsic facet integrity; and (iii) evaluating the patient’s overall physiological suitability for potentially more demanding fusion procedures. When these criteria are met, facet-sparing uniportal endoscopic decompression alone may offer a compelling alternative, providing effective symptom relief without the higher risks and burdens inherent in fusion. Conversely, patients with clear dynamic instability, those requiring extensive facetectomy that would compromise intrinsic stability, or those whose primary complaint is mechanical back pain were outside the scope of this study and would likely still necessitate fusion.

Limitations inherent to this case series include the small sample size, short follow-up duration, inherent selection bias, and the absence of a direct comparator group treated with fusion. These factors, coupled with the operator-dependent nature of endoscopic spine surgery performed by an experienced endoscopic surgeon at a single tertiary centre, limit the immediate generalizability of these results.

Nevertheless, the consistently positive preliminary outcomes provide a strong encouragement for further investigation. To provide higher-level evidence and definitively compare outcomes to the standard of care, a prospective, multicentre comparative study assessing decompression alone versus fusion in this specific elderly, high-grade spondylolisthesis population is essential. Confirming the durability of these results necessitates long-term follow-up exceeding 5 years, ideally incorporating dynamic imaging studies. Furthermore, the successful broader adoption of such advanced endoscopic techniques will depend on the development and establishment of standardized training programs.

In conclusion, this case series suggests that meticulous patient selection coupled with facet-preserving uniportal endoscopic decompression holds promise, achieving excellent short-term outcomes in elderly patients with stable, higher-grade spondylolisthesis. While spinal fusion has traditionally been the preferred method to achieve stability in such cases [3], this minimally invasive approach offers a potential strategy to reduce the need for fusion, aligning well with personalized care and quality-of-life enhancement in an aging population.
